# Prolonged Maternal Zika Viremia as a Marker of Adverse Perinatal Outcomes

**DOI:** 10.3201/eid2702.200684

**Published:** 2021-02

**Authors:** Léo Pomar, Véronique Lambert, Séverine Matheus, Céline Pomar, Najeh Hcini, Gabriel Carles, Dominique Rousset, Manon Vouga, Alice Panchaud, David Baud

**Affiliations:** Lausanne University Hospital, Lausanne, Switzerland (L. Pomar, C. Pomar, M. Vouga, A. Panchaud, D. Baud);; Centre Hospitalier de l’Ouest Guyanais Franck Joly, Saint-Laurent-du-Maroni, French Guiana (V. Lambert, C. Pomar, N. Hcini, G. Carles);; Institut Pasteur, Paris, France (S. Matheus);; Institut Pasteur of French Guiana, Cayenne, French Guiana (S. Matheus, D. Rousset);; University of Bern, Bern, Switzerland (A. Panchaud)

**Keywords:** Zika, prolonged viremia, congenital infection, congenital Zika syndrome, viruses, mosquitoborne diseases, vector-borne infections, French Guiana, Zika virus, ZIKV

## Abstract

Whether prolonged maternal viremia after Zika virus infection represents a risk factor for maternal–fetal transmission and subsequent adverse outcomes remains unclear. In this prospective cohort study in French Guiana, we enrolled Zika virus–infected pregnant women with a positive PCR result at inclusion and noninfected pregnant women; both groups underwent serologic testing in each trimester and at delivery during January–July 2016. Prolonged viremia was defined as ongoing virus detection >30 days postinfection. Adverse outcomes (fetal loss or neurologic anomalies) were more common in fetuses and neonates from mothers with prolonged viremia (40.0%) compared with those from infected mothers without prolonged viremia (5.3%, adjusted relative risk [aRR] 7.2 [95% CI 0.9–57.6]) or those from noninfected mothers (6.6%, aRR 6.7 [95% CI 3.0–15.1]). Congenital infections were confirmed more often in fetuses and neonates from mothers with prolonged viremia compared with the other 2 groups (60.0% vs. 26.3% vs. 0.0%, aRR 2.3 [95% CI 0.9–5.5]).

The recent worldwide epidemic confirmed maternal–fetal transmission of Zika virus (ZIKV) and its association with adverse perinatal outcomes, particularly severe central nervous system lesions and fetal losses (*1*–*3*). Whether prolonged viremia after ZIKV infection in pregnant women represents a risk factor for maternal–fetal transmission, congenital Zika syndrome (CZS), or other adverse outcomes is on ongoing controversy (*4*).

ZIKV is detectable in maternal blood by reverse transcription PCR (RT-PCR) during the acute phase of infection. ZIKV viremia usually lasts from 2 days before to 16 days after symptom onset; median time of ZIKV RNA clearance is 5 days (*5*). Driggers et al. (*6*) detected ZIKV RNA in maternal serum samples 8 weeks after onset of clinical symptoms; they suggested that prolonged viremia might occur as a consequence of viral replication in the fetus or placenta and might be correlated with CZS. In other reports, however, prolonged maternal viremia has been described in pregnant women with both normal and adverse fetal outcomes (*6*–*10*).

In a cohort of pregnant women exposed to ZIKV in French Guiana, we investigated the impact of prolonged viremia on fetal and neonatal adverse outcomes (fetal loss or neurologic anomalies) compared with infected pregnant women without prolonged viremia and noninfected pregnant women. We also compared the rates of congenital infections between these groups.

## Methods

### Study Population, Recruitment, and Follow-up

The study was conducted at the Centre Hospitalier de l’Ouest Guyanais (CHOG; Saint-Laurent-du-Maroni, in French Guiana) during January 1–July 15, 2016, at the beginning of the ZIKV epidemic. Persons for inclusion were initially identified either through routine serologic testing of all pregnant women admitted to the prenatal diagnosis unit of CHOG (performed in each trimester of pregnancy and at birth), or through serologic and molecular testing in cases of maternal symptoms, acute exposure in the previous 2 weeks (patients who arrived in French Guiana from a nonendemic country or patients who arrived from an endemic country [e.g., Brazil in January 2016] before the epidemic began in French Guiana), fetal or neonatal central nervous system anomalies, or in cases of amniocentesis performed for suspected CZS or other indications (i.e., aneuploidy diagnosis). All pregnant women with ZIKV testing available with ongoing follow-up at CHOG were invited to participate in the study. Written consent was obtained from all participants. The study received ethics approval from the CHOG institutional review board (*11*). Data regarding demographic, medical, and obstetrical characteristics and possible risk factors for congenital diseases were collected prospectively at inclusion.

Patients were monitored in accordance with the clinical standard of care in France, with the exception that prenatal ultrasound was performed monthly for patients who tested positive for ZIKV. Two supplementary ultrasounds were provided to patients who tested negative for ZIKV (at 26–28 and 36–38 weeks’ gestation), as recommended by health authorities in France and other organizations (*12*–*15*). In cases of fetal loss (>14 weeks’ gestation) or termination of pregnancy, a postmortem examination was offered, including macroscopic imaging and anatomic–pathologic examination.

In cases of live birth, a clinical examination (with particular attention to neurologic and systemic symptoms such as hypertonia, swallowing disorders, hypotonia, hepatomegaly, and jaundice) and testing for congenital ZIKV infection were performed for all neonates. Biologic, ophthalmologic, and imaging follow-up was offered for neonates from ZIKV-infected pregnant women (*16*).

Pregnant women not followed at the CHOG prenatal diagnosis unit after ZIKV testing, as well as patients who only delivered at CHOG without appropriate prenatal follow-up, were excluded from this study. Fetuses or neonates who did not undergo testing for ZIKV at birth or an appropriate postnatal or postmortem examination also were excluded.

### Definition of Maternal ZIKV Infection

Molecular and serologic testing was performed at the French Guiana National Reference Center for arboviruses (Institut Pasteur of French Guiana, Cayenne, French Guiana) using the Realstar Zika Kit (Altona Diagnostics GmbH, https://altona-diagnostics.com) for RT-PCR, in-house IgM and IgG antibody-capture ELISA, and microneutralization assays for serologic testing. The limit of detection for serum samples tested using the Realstar Zika Kit was 0.61 (95% CI 0.39–1.27) copies/μL (*17*). A cycle threshold (C_t_) value <37 was considered positive.

When ZIKV RNA was initially detected, molecular diagnosis was performed monthly and at delivery on maternal serum samples. Prolonged viremia was defined as ongoing viral detection >30 days after symptom onset or after initial detection of viremia in asymptomatic patients. Absence of prolonged viremia in infected patients was defined as a subsequent negative molecular test <30 days after symptom onset or after initial detection of viremia in asymptomatic patients.

In all cases, ZIKV serologic tests were performed in each trimester of pregnancy and at delivery. Patients with only positive IgM without RT-PCR testing or with a negative RT-PCR result were excluded from the analysis.

Maternal symptoms potentially related to ZIKV were recorded at each prenatal visit and at birth. These symptoms included rash, fever, asthenia, pruritus, arthralgia, retro-orbital headache, myalgia, conjunctival hyperemia, edema of the extremities, and neurologic complications (*18*). Asymptomatic pregnant women who remained ZIKV-negative on serologic tests during their pregnancy and at delivery were considered noninfected.

### Definition of Fetal and Neonatal Adverse Outcomes and Congenital Infection 

Fetal and neonatal outcomes were reviewed by 3 independent reviewers, including 2 maternal–fetal medicine specialists who had not been in contact with these patients previously. Cerebral anomalies were defined as >1 major cerebral sign, based on an extended definition of CZS (*14*,*19*,*20*) (Appendix 1 Table 1). Fetal loss was defined as a spontaneous fetal demise at >14 weeks’ gestation, including late miscarriages (14–24 weeks’ gestation) and stillbirths (fetal demise >24 weeks’ gestation up to intrapartum); intrapartum and early postpartum deaths were excluded. For the analysis, fetuses and neonates who had major cerebral anomalies, fetal losses, or both were categorized as having adverse outcomes. Termination of pregnancy for reasons other than major cerebral abnormalities were not considered as adverse outcomes in this analysis. 

All fetuses and neonates underwent ZIKV testing at birth or after fetal loss. Prenatal testing by amniocentesis was offered in those with fetal anomalies, if an amniocentesis was indicated for other indications (i.e., aneuploidy diagnosis), or both. A confirmed congenital ZIKV infection was defined either by ZIKV RNA amplification by RT-PCR from >1 fetal or neonatal sample (e.g., placenta, amniotic fluid, cerebrospinal fluid, urine, or blood) or identification of ZIKV-specific IgM in the umbilical cord or neonatal blood or in cerebrospinal fluid. Details of congenital ZIKV testing are discussed elsewhere (*19*).

### Statistical Analyses

Standardized differences were calculated to compare baseline characteristics of patients with prolonged viremia to those of the reference groups (i.e., pregnant women with positive RT-PCR results without prolonged viremia and noninfected pregnant women). These characteristics were considered unbalanced when the standardized difference was >0.15.

Relative risks (RRs) and 95% CIs were calculated for fetal and neonatal adverse outcomes by using generalized linear regression. Adjusted RRs (aRRs) were calculated for variables reflecting unbalanced baseline characteristics that could represent confounding factors (maternal age and maternal underlying conditions). When fetuses from mothers with prolonged viremia were compared with those from infected mothers without prolonged viremia, RRs were also adjusted for the trimester of maternal infection diagnosis. A robust SE option was used for twins in order to not affect the variance in considering twins as separate cases.

We conducted a sensitivity analysis to test the robustness of our findings, using different criteria for the diagnosis of maternal prolonged viremia. We compared fetuses and neonates from patients with stable or increasing quantitative PCR (qPCR) values between the inclusion and the first follow-up with those from mothers with decreasing qPCR values.

The missing data were considered to be random, and thus we performed a complete case analysis. All statistical analyses were conducted by using Stata 15 (StataCorp, https://www.stata.com).

## Results

### Recruitment and Maternal ZIKV Diagnosis

**Figure 1:**
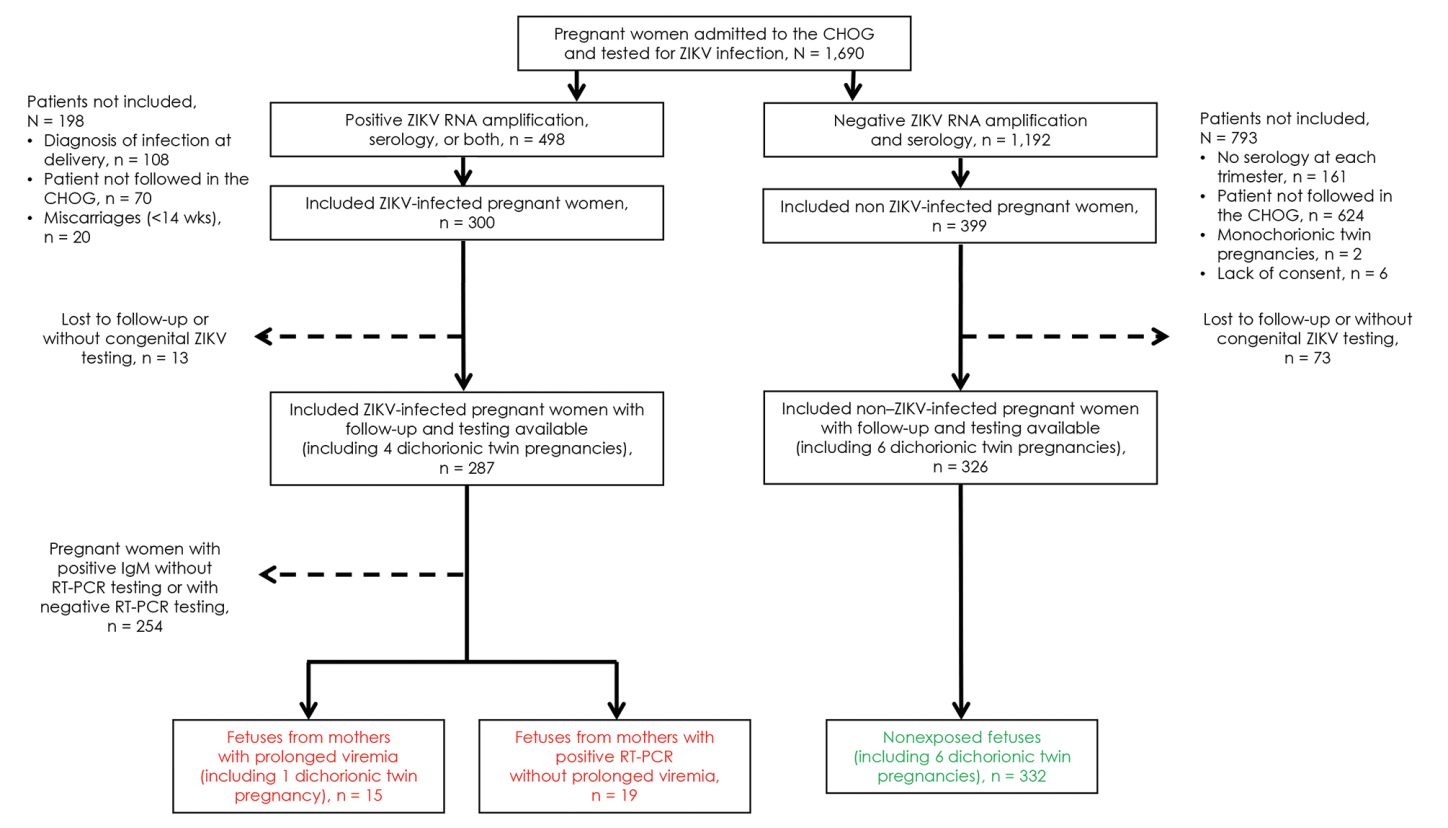
Flowchart of pregnant women admitted to CHOG, French Guiana, January 1–July 15, 2016. All women were routinely tested for ZIKV-specific IgM and IgG in each trimester of pregnancy and at delivery. In cases of maternal symptoms, acute exposure in the previous 2 weeks, fetal anomalies, or if an amniocentesis was indicated, pregnant women were also tested for ZIKV RNA by RT-PCR in blood and urine. Patients with a positive RT-PCR result were offered to participate in the study and underwent monthly RT-PCR testing up to clearance or delivery. Prolonged viremia was defined as ongoing viral detection >30 days after symptom onset or after initial detection of viremia. Asymptomatic patients who remained negative for ZIKV IgM during the whole pregnancy were recruited and considered as non–ZIKV-infected. Patients with only positive IgM without or with a negative RT-PCR test result were excluded of this analysis because of the inability to accurately date the onset and clearance of viremia. Patients without appropriate monthly follow-up were also excluded from this study (e.g., those who had early miscarriages, late diagnosis of infection at delivery, or were not followed in our center after the diagnosis). After expulsion, fetal losses were tested by RT-PCR (as well as by IgM, if available). Fetuses with anomalies were tested by RT-PCR on amniotic fluid. Neonates were tested for ZIKV at birth (RT-PCR on placenta, urine, blood and IgM on blood [as well as on cerebrospinal fluid, if symptomatic]). Fetuses and neonates without appropriate testing and examination after fetal loss or birth were excluded from this analysis. Overall, 15 fetuses from 14 infected pregnant women with prolonged viremia (including 1 with a twin pregnancy), 19 fetuses from 19 infected pregnant women without prolonged viremia, and 332 fetuses from 326 noninfected pregnant women (including 6 with twin pregnancies) were included. CHOG, Centre Hospitalier de l’Ouest Guyanais (Saint-Laurent-du-Maroni, French Guiana); RT-PCR, reverse transcription PCR; ZIKV, Zika virus.

During January 1–July 15, 2016, a total of 1,690 pregnant women were admitted to CHOG and tested for ZIKV infection (Figure 1). Among 498 women with a positive test, 198 were not prospectively followed in the CHOG prenatal diagnosis unit (including 20 patients with early miscarriages, 70 who were followed elsewhere after initial diagnosis of ZIKV infection, and 108 with a diagnosis at delivery without appropriate prenatal follow-up). A total of 300 pregnant women (including 5 with dichorionic twin pregnancies) with a positive ZIKV test were monitored in the unit. Full fetal and neonatal testing and follow-up was available for 287 of them (including 4 with twin pregnancies). Among these 287 ZIKV-positive patients, 254 (including 3 with twin pregnancies) had ZIKV infection diagnosed only by positive IgM, without molecular testing or with negative molecular testing, preventing calculation of the start of viremia. These patients were excluded from the analysis. Positive molecular testing was found in 33 patients (including 1 with a twin pregnancy); 30 were positive by RT-PCR in maternal blood, 9 were positive by RT-PCR in urine samples. Among these ZIKV RNA–positive pregnant women, 14 (including 1 with a twin pregnancy) exhibited a prolonged viremia, whereas the other 19 became subsequently negative within 30 days. Details of positive molecular testing are presented in Appendix 1 Table 2 and the evolution of qPCR values for each positive patient in Appendix 2 Figure.

During the same period, 399 pregnant women (including 6 with dichorionic twin pregnancies) with negative serologic test results for ZIKV were followed for routine scans at CHOG. Full maternal, placental, and neonatal testing was available for 326 of them (including 6 with twin pregnancies). These patients remained negative for ZIKV during the entire pregnancy and at delivery, and constituted the noninfected group. The recruitment process is summarized in Figure 1.

### Baseline Characteristics of Participants

As shown in Appendix 1 Table 3, baseline characteristics were similar between pregnant women with prolonged viremia and the reference groups, except for a history of congenital abnormalities or intrauterine fetal demise, maternal underlying conditions, rate of dichorionic twins, and high risk for fetal aneuploidy (>1/250), for which standardized differences >0.15 were observed. Maternal ZIKV infections were diagnosed earlier in pregnancies with prolonged viremia than in those without prolonged viremia.

### Fetal and Neonatal Adverse Outcomes and Congenital Infections

**Figure 2:**
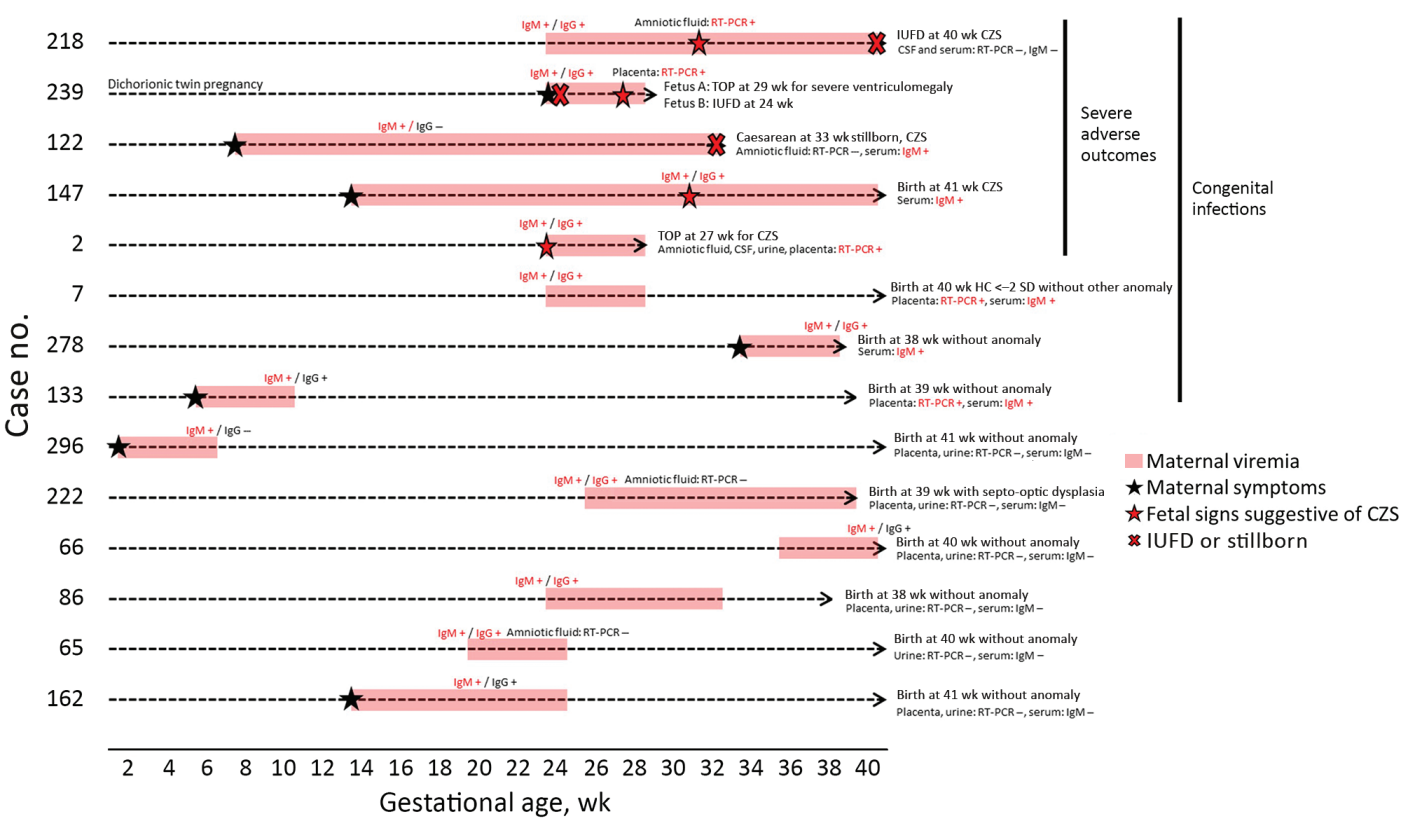
Pregnancy outcomes of patients with prolonged viremia in a cohort study of pregnant women admitted to Centre Hospitalier de l’Ouest Guyanais, French Guiana, January 1–July 15, 2016. Description and outcomes of 14 pregnancies with prolonged maternal ZIKV viremia (including 1 with dichorionic twin pregnancy). Congenital ZIKV infection was confirmed in 9/15 (60%) fetuses, and 6/15 (40%) fetuses had adverse outcomes. Four of them had multiple abnormalities consistent with CZS (1 live birth, 1 TOP, 1 IUFD, and 1 stillbirth). The 2 fetuses from the dichorionic twin pregnancy also had adverse outcomes, with an IUFD at 24 wk and a TOP for severe ventriculomegaly in the other fetus at 29 wk. CSF, cerebrospinal fluid; CZS, congenital Zika syndrome; HC, head circumference; IUFD, intrauterine fetal demise; RT-PCR, reverse transcription PCR; TOP, termination of pregnancy; ZIKV, Zika virus.

**Figure 3:**
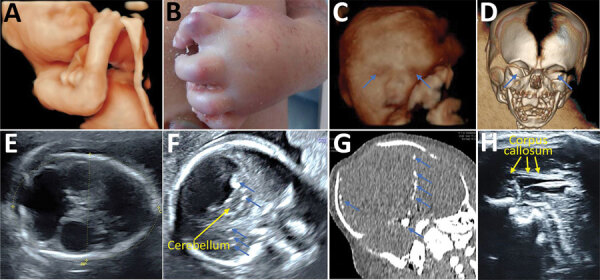
Prenatal ultrasound, computed tomography, and postmortem aspect of a fetus with congenital Zika syndrome related to maternal prolonged viremia in patient (case no. 122) in a cohort study of pregnant women admitted to Centre Hospitalier de l’Ouest Guyanais, French Guiana, January 1–July 15, 2016. The mother had symptomatic acute Zika virus infection at 8 weeks’ gestation (and had ongoing viremia until birth of her stillborn child with signs of congenital Zika syndrome. Severe microcephaly, ventriculomegaly, and calcifications were detected by ultrasound at 13 weeks’ gestation. Overall, this fetus had arthrogryposis detected on 3-D ultrasound (A) and postmortem (B); severe bilateral microphthalmia (blue arrows) detected on 3-D ultrasound (C) and fetal computed tomography (D); microcephaly with atrophic cortex detected on ultrasound (E) showing a head circumference of 160 mm at 25 weeks’ gestation (−5 SDs); ventriculomegaly detected on ultrasound (E); brain calcifications (blue arrows) detected on ultrasound (F) and computed tomography (G); pontocerebellar hypoplasia (yellow arrows) detected on ultrasound (F); and corpus callosum dysgenesis (yellow arrows) detected on ultrasound (H).

ZIKV testing and outcomes were available for 15 fetuses from 14 infected pregnant women with prolonged viremia (including 1 with a dichorionic twin pregnancy) (Appendix 1 Tables 4, 5). Two pregnancies (2/15 [13.3%]) were terminated for severe neurologic anomalies, and 2 fetal losses (2/15 [13.3%]) were recorded. Among fetuses with imaging studies and examinations available, 4 (4/14 [28.6%]) exhibited neurologic anomalies and 4 (4/11 [36.4%]) ocular anomalies (Appendix 1 Table 4). Congenital ZIKV infections were confirmed in 9 (9/15 [60.0%]) of these fetuses or newborns, of which 6 (6/9 [66.7%]) cases resulted in adverse outcomes (4 suspected CZS and 2 fetal losses). All pregnancy outcomes for women with prolonged viremia are detailed in Figure 2. Figure 3 presents an example of CZS related to maternal prolonged viremia.

Among 19 fetuses from mothers with an initially positive RT-PCR result without prolonged viremia, no fetal loss or termination of pregnancy was recorded. Neurologic anomalies were found in 1 (1/19 [5.3%]) of these fetuses, who also had ocular anomalies (confirmed at birth). Congenital ZIKV infections were confirmed in 5 (5/19 [26.3%]) of these newborns, of which 1 (1/5 [20%]) resulted in adverse outcomes (suspected CZS).

ZIKV testing and outcomes were available for 332 fetuses or newborns from noninfected pregnant women (including 6 with dichorionic twin pregnancies). Four pregnancies (4/332 [1.2%]) resulted in termination of pregnancy (1 for severe neurologic anomalies and 3 for extraneurologic anomalies [congenital heart disease, skeletal dysplasia, and Down syndrome]). Four (4/332 [1.2%]) fetal losses were recorded. Among the fetuses with imaging studies and examination available, 17 (17/331 [5.1%]) had neurologic anomalies. None of these fetuses or neonates were found to be ZIKV-positive at birth.

### Associations between Prolonged Viremia, Fetal and Neonatal Adverse Outcomes, and Congenital Infections

Overall, fetuses or neonates from mothers with a prolonged viremia during pregnancy exhibited a higher risk for adverse outcomes (6/15 [40%] with fetal loss, neurologic anomalies, or both) compared with those from infected mothers without prolonged viremia (1/19 [5.3%], RR 7.6 [95% CI 1.0–56.5]) and noninfected mothers (20/332 [6.0%], RR 6.6 [95% CI 3.1–14.1]). Similar results were observed for fetal losses and neurologic anomalies when analyzed independently (Appendix 1 Table 4). After adjustment for maternal underlying conditions and considering the twin pregnancies in the variance, these associations and trends persisted (aRR 7.2 [95% CI 0.9–57.6] when compared with fetuses from infected mothers without prolonged viremia and aRR 6.7 [95% CI 3.0–15.1] when compared with fetuses from noninfected mothers). In the comparison with fetuses from infected mothers without prolonged viremia, the analysis was also adjusted for the trimester of maternal infection diagnosis (Appendix 1 Tables 4, 6).

Congenital infections were confirmed more frequently in fetuses from mothers with prolonged viremia (9/15 [60.0%]) when compared with those from infected mothers without prolonged viremia (5/19 [26.3%], RR 2.3 [95% CI 1.0–5.4]) and noninfected mothers (0/332 [0.0%]). After adjustment for the trimester of maternal infection diagnosis and consideration of twin pregnancies in the variance, this trend persists (aRR 2.3 [95% CI 0.9–5.5]) (Appendix 1 Tables 4, 6).

Our sensitivity analysis found similar results when considering the evolution of qPCR values between the inclusion and the first follow-up instead of prolonged viremia as a binary variable (Appendix 1 Table 7). Neurologic or systemic symptoms at birth were also more frequent in newborns from mothers with prolonged viremia compared with those from infected mothers without prolonged viremia or non-infected mothers (Appendix 1 Table 5).

## Discussion

The main findings of this cohort study are 2-fold. First, maternal ZIKV infection with prolonged viremia is associated with a 7-fold increased risk for fetal or neonatal adverse outcomes compared with pregnancies without prolonged viremia. Second, maternal prolonged viremia is associated with a 2-fold increased risk for confirmed congenital infection compared with infected mothers without prolonged viremia.

Our study was limited by the sample size of the infected group because only patients with a positive ZIKV RT-PCR result at enrollment were included in the analysis and followed up with monthly RT-PCR testing until clearance or delivery. Because the measured effect size was high, the sample size was sufficient to identify an association. The limited number of cases with prolonged viremia, however, forced us to group all adverse outcomes together to conduct an analysis with sufficient power and prevented the evaluation of its association with individual signs or symptoms or new characteristics (*21*,*22*).

Although ZIKV testing was based on the previous guidelines relevant during the 2015–2016 epidemic with adaptation to local capacities, testing does not follow the more recent CDC guidelines (*23*) (i.e., patients considered as noninfected underwent serologic testing in each trimester rather than nucleic acid testing, as is currently recommended). Patients included as noninfected, however, remained negative for IgM and IgG in each trimester and at delivery, limiting the risk for exposure misclassification. Similarly, women with only positive IgM testing were excluded from the study because some of them might have had undetected prolonged viremia, which would have led to an exposure misclassification and an underestimation of the consequences of prolonged viremia.

Information about the sensitivity and specificity of neonatal testing remains limited, and several studies have shown the progressive disappearance of ZIKV RNA in the maternal–fetal compartments (*24*). Although the identification of IgM in fetal or neonatal blood was used to avoid congenital ZIKV false negatives, we cannot exclude an outcome misclassification because some neonates with negative results could have been infected by ZIKV without viremia and immunity against ZIKV detectable at birth. This risk is likely low given that >80% of fetuses or neonates from infected pregnant women underwent testing in >3 different samples (including blood, urine, placenta, cerebrospinal, and amniotic fluid) (*19*), and all neonates from noninfected pregnant women underwent serologic testing at birth. Undetected congenital infections in the 2 reference groups might result in overestimation of the effect of prolonged viremia overall.

Neonates from noninfected mothers underwent postnatal transfontanellar ultrasound (as well as by computed tomography and magnetic resonance imaging, if available) only in the case of an abnormal prenatal ultrasound or symptoms at birth, in contrast to those from infected mothers who underwent routine postnatal imaging. However, when they are asymptomatic, some neonates from noninfected mothers might have undetected cerebral anomalies at birth, resulting in an overestimation of the consequences of prolonged Zika viremia. We cannot totally exclude this bias; however, all neonates from noninfected mothers underwent multiple prenatal ultrasound assessments (enhanced by 2 supplementary examinations with neurosonograms during the epidemic), reducing the risk for undetected cerebral anomalies.

Molecular testing has been proposed for use at different stages of pregnancy depending on the presence of maternal symptoms. Thus, symptomatic patients might have had ZIKV infection diagnosed earlier than asymptomatic patients for whom a molecular diagnosis was proposed in cases of fetal anomalies, amniocentesis (for fetal signs or other indications [i.e., aneuploidy diagnosis]), or both, occurring later in pregnancy. Among infected pregnant women without prolonged viremia, 7 were asymptomatic with a continuous exposure and did not have molecular testing before amniocentesis, preventing accurate identification of the time of infection during pregnancy. Thus, we cannot exclude that some of these patients were in fact infected earlier in pregnancy and had an undetected prolonged viremia resulting in an exposure misclassification. This bias would result in an underestimation of the consequences of prolonged viremia because a case that included neurologic anomalies (CZS) in the reference group from infected mothers without prolonged viremia could in fact be related to prolonged viremia. Similarly, we cannot exclude a potential selection bias given that some patients were tested by RT-PCR because their fetus had anomalies at inclusion. Because this proportion did not differ between the groups (3/14 vs. 4/19), even if we consider only anomalies suggestive of fetal infection at inclusion (2/14 vs. 2/19), we would not expect relative risks to be significantly affected. However, this potential selection bias could overestimate absolute frequencies of fetal anomalies in RT-PCR–positive patients, and this bias seems to be inherent in contemporary cohorts because inclusion after the observation of fetal anomalies potentially related to Zika were common.

Driggers et al. (*6*) were the first to highlight a possible association between prolonged maternal viremia and congenital infection with CZS. In their cohort study, Rodo et al. (*25*) described 9 cases of prolonged maternal viremia, among which 2 resulted in congenital ZIKV infection, with 1 of those 2 infections resulting in severe neurologic anomalies. The rates of congenital infection and fetal or neonatal adverse outcomes in women with prolonged viremia seem to be higher in our study (9/15 for congenital infections and 4/15 for CZS). This difference might be explained by the exclusive use of amniocentesis for diagnosis in the Rodo et al. cohort, whereas multiple fetal or neonatal samples were tested in our study (>80% of the fetuses or newborns had >3 different samples tested). Our results are congruent with Meaney et al. (*10*), who identified prolonged ZIKV RNA detection in 4 symptomatic pregnant women in the US Zika Pregnancy Registry, of which 1 pregnancy (25%) resulted in congenital Zika syndrome. Suy et al. (*8*) described a case of CZS with prolonged maternal viremia where the viral load in the maternal serum sample remained stable for 14 weeks and then became negative, instead of decreasing progressively, as would be expected. Suy et al. suggested that the prolonged viremia that was detected in the mother could be the result of viral replication in the fetus or placenta, which thus might act as a reservoir. However, their study still lacks a consensual threshold to define prolonged viremia. In our study, we defined prolonged viremia as ongoing viral detection >30 days after symptom onset or after initial detection of viremia for a question of feasibility. Indeed, many of our patients were living around the Maroni River, in isolated areas, and came monthly to CHOG for their clinical follow-up. In light of this geographic distance, we decided that monthly RT-PCR testing in case of initial detection of viremia was the most appropriate. In the context of a smaller area with local facilities, testing patients every 2 weeks to fulfill the threshold used in other studies might have been useful (*10*,*25*).

Negative and positive predictive values of prolonged maternal viremia for congenital infections and adverse outcomes related to ZIKV seem to be moderate because fetal and neonatal adverse outcomes and congenital infections also occur in pregnant women without identified prolonged viremia. One explanation could be that prolonged viremia might reflect viral replication in the placenta without further involvement for the fetus (*27*). In addition, some of our cases with prolonged maternal viremia (6/15) did not exhibit congenital infections, suggesting that prolonged maternal viremia might also reflect persistent viral replication in other reservoirs than the fetus or the placenta. The study of Rodo et al. (*10*) and the CDC report (*25*) also described fetuses without congenital infection or adverse outcomes from mothers with prolonged viremia (*10*,*25*).

Our results also indicate that noninfected women exhibited a 5.1% risk for fetal neurologic anomalies and 1.2% risk for fetal losses (higher than the estimation of 3% risk for neurologic anomalies and 0.5%–1.0% risk for fetal losses in developed countries), reflecting that other etiologies for adverse perinatal outcomes remain present even in the context of a ZIKV epidemic (*28*), particularly in French Guiana, where pregnant women are exposed to lead poisoning, poverty, and higher risk for underlying conditions (*29*). To reduce the impact of these cofounding factors on our assessment of adverse neonatal outcomes related to prolonged ZIKV viremia, we chose to adjust the RR estimates for unbalanced maternal underlying conditions that might have an effect on the exposure and on adverse perinatal outcomes.

In conclusion, prolonged maternal ZIKV viremia could be a marker for an increased risk for maternal–fetal transmission and subsequent adverse perinatal outcomes. Even if prolonged maternal viremia is not consistently present in cases of congenital infection, it might reflect active viral replication in the fetal–placental compartment and should lead to an enhanced prenatal and neonatal follow-up.

Appendix 1 Additional information on prolonged maternal Zika viremia as a marker of adverse perinatal outcomes.

Appendix 2 Additional figure for study of prolonged maternal Zika viremia as a marker of adverse perinatal outcomes.
