# Supplementary material for: Prolonged Maternal Zika Viremia as a Marker of Adverse Perinatal Outcomes
Source: Emerg Infect Dis. 2021 Feb;27(2):490–8. doi: 10.3201/eid2702.200684 (PMC7853546; doi:10.3201/eid2702.200684)
Supplement: Appendix 2 — Additional figure for study of prolonged maternal Zika viremia as a marker of adverse perinatal outcomes. [file 20-0684-Techapp-s2.pdf]

Article DOI: https://doi.org/10.3201/eid2702.200684

Prolonged Maternal Zika Viremia as a Marker of Adverse Perinatal Outcomes

Appendix 2

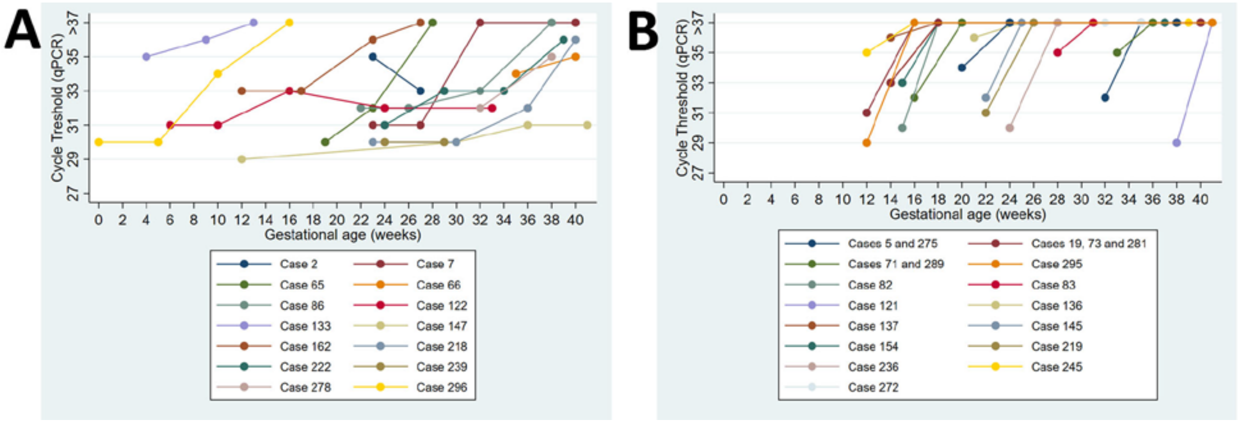

Appendix 2 Figure. Evolution of qPCR values (cycle threshold) are presented, according to gestational age at sampling, for each patients. Left: patients with prolonged viremia (positive PCR >30 days after initial detection); Right: Zika-infected patients without prolonged viremia (positive PCR at inclusion, with negative PCR at the first follow-up ≤30 days after the inclusion). Prolonged maternal Zika viremia study, French Guiana, 2016–2017.
